# Effect of 6-week retro or forward walking program on pain, functional disability, quadriceps muscle strength, and performance in individuals with knee osteoarthritis: a randomized controlled trial (retro-walking trial)

**DOI:** 10.1186/s12891-019-2537-9

**Published:** 2019-04-09

**Authors:** Ahmad H. Alghadir, Shahnawaz Anwer, Bibhuti Sarkar, Ashis K. Paul, Dilshad Anwar

**Affiliations:** 1 Rehabilitation Research Chair, College of Applied Medical Sciences, King Saud University, P.O. Box-10219, Riyadh, 11433 Saudi Arabia; 2 Deparment of Building and Real Estate, Hong Kong Polytechnic University, Kowloon, Hong Kong Special Administrative Region China; 3 National Institute for the Locomotor Disabilities (Divyangjan), Kolkata, India; 4 Anand Vihar Hospital, Mahanadi Coalfields Limited, Sambalpur, Odisha India; 5 Bone Joint and Trauma Clinic, Darbhanga, India

**Keywords:** Knee osteoarthritis, Walking, Exercise, Retro walking, Muscle strength

## Abstract

**Background:**

Previous studies reported the beneficial effects of walking in individual with mild to moderate knee osteoarthritis (OA). The current study aimed to compare the effect of 6-week retro versus forward walking program versus control group on pain, functional disability, quadriceps muscle strength and physical performance in individuals with knee OA.

**Methods:**

A three-arm single-blinded, randomized, controlled trial and intention-to-treat analysis was conducted in outpatient physiotherapy department, King Saud University, Saudi Arabia. Sixty-eight individuals (mean age, 55.6 years; 38 female) with knee OA participated. The participants in the retro or forward walking group completed 10 min of supervised retro or forward walking training in addition to usual care, 3 days/week for 6 weeks**.** The control group received a routine physiotherapy program. This program comprises a combination of closed and open kinematic chain exercises, including straight leg raising, isometric quadriceps, isometric hip adduction, terminal knee extension, semi-squat, and leg press. The primary outcomes were mean pain and knee function score measured by the numerical rating scale and the Western Ontario and McMaster Universities Osteoarthritis Index, respectively. The secondary outcomes were mean score of quadriceps muscle strength and timed up and go test scores. All the outcomes were analyzed at baseline and week 6.

**Results:**

In total, 68 subjects participated in this 6-week randomized, controlled trial. The completion rates of the primary and secondary outcome measures at week 6 were 91, 87, and 82% in the retro walking, forward walking, and control groups, respectively. In the intention-to-treat analysis, the retro walking group had a greater reduction in pain intensity (mean changes, 1.8 versus 1; *p* = 0.01) and functional disability (mean changes, 4.8 versus 2.2; *p* = 0.008) than the control group. Similarly, the retro walking group had a greater improvement in the quadriceps muscle strength (mean changes, 1.7 kg versus 0.7 kg; p = 0.008) and the timed up and go test (mean changes, 0.6 s versus 0.1 s; *p* = 0.003) than the control group.

**Conclusions:**

The 6-week retro walking program compared with forward walking or control groups resulted in greater reduction in pain and functional disability and improved quadriceps muscle strength and performance in individuals with knee OA.

**Trial registration:**

Controlled Trials ISRCTN12850845, Registered 26 January 2015.

## Background

The prevalence of osteoarthritis (OA) is gradually increasing in both low- and high-income countries [1]. The Global Burden of Disease studies recently indicated that knee OA is the fastest increasing major health disorder and the second global cause of disability [2]. In the lower extremity, the knee is often affected [3], and knee OA results in significant mobility restrictions [4] and a substantial financial burden [5]. The risk of OA-associated disability is equal to that of cardiac disorders [6] and more common than any other medical problem in older populations [7]. The common clinical manifestations of knee OA include pain, stiffness, joint enlargement, crepitus, muscle weakness, deformity, impaired proprioception, reduced joint motion, and disability [8]. Therapeutic exercises are often used to improve physiological impairments such as reduced joint motion, muscle weakness, impaired balance, disability, and proprioception [9, 10].

In open kinetic chain exercises, the distal segment of the lower limb is free to move during movement. In contrast, the distal segment of the lower limb is fixed during the closed kinetic chain exercises. The closed kinetic chain exercises are closely related to the activities that we performed in daily life and more functional in nature [11, 12]. In addition, closed kinetic chain exercise could also improve joint proprioception, muscle strength, and balance [8, 13–15]. Walking is a closed kinetic chain exercise program which allows initiation of weight bearing and early mobilization in knee rehabilitation. Regular walking exercises are beneficial, and it is recommended to reduce pain and disability in people with knee OA [16–18]. A moderate effect of walking compared to home-based quadriceps strengthening exercises on knee pain and function was reported in the previous systematic review and meta-analysis [19]. Kovar et al. [20] reported improved function and no worsening of OA related symptoms after supervised fitness walking program compared to patient education program.

Most of the previous studies investigated the effects of backward running, only few studies presented the effects of backward walking. Previous studies have suggested that the backward running program may give additional benefits more than those experienced by forward running in healthy adults [21, 22]. Retro walking is considered an effective closed kinetic chain exercise to improve lower muscles strength and the equilibrium of the human body [23]. Another study reported that backward running causes reduced eccentric activity of the quadriceps, while concentric and the isometric quadriceps activity was preserved [24]. More recently, Loew et al. [25] reported improved pain relief and aerobic fitness level, without aggravating symptoms following walking programs in patients with knee OA. However, Loew et al. [25] did not compare effects in pain and aerobic fitness level with the control group. Significant improvement in function after 3 weeks of retro walking in addition to a routine physiotherapy in person with knee OA was previously noted [26]. Moreover, a recent phase I trial indicated that 70 min walking per week was safe, feasible, and tolerated by people with severe knee OA; however, longer walking-periods may exacerbate knee pain levels [27]. Furthermore, a phase II randomized controlled trial reported improved cardiovascular health without reducing knee pain following a 12-week walking program in people with severe knee OA [28]. In contrast, worsening of OA-related symptoms after a walking program in person with knee OA was also reported [29]. Thus, we hypothesized that a less intensive walking program such as retro walking program could provide an additional benefit more than those experienced by forward walking program in the previous studies. Therefore, the primary aim of the present study was to compare the effect of retro versus forward walking versus control group on knee pain and function in people with knee OA. The secondary aims were to compare the effect of retro versus forward walking versus control group on quadriceps muscle strength and performance in people with knee OA.

## Methods

### Trial design

This Retro-walking trial was a three-arm single-blinded randomized controlled trial comparing retro walking, forward walking, and control. The participants were randomly assigned to retro walking, forward walking, or control groups. Blank folders were numbered from 1 to 68, given concealed codes for the group assignment by an independent therapist, and kept in a safe locker. When a participant was eligible and agree to participate, an independent therapist drew the next folder from the file to decide the group assignment. All participants received their assigned intervention in the outpatient physiotherapy department. The Declaration of Helsinki was followed for all experiments. Recruitment of the participants took place from August 3, 2014 through October 30, 2015, and the trial ended on December 30, 2015. Participants were requested to sign a written informed consent form approved by the institution ethics committee of King Saud University. The CONSORT guidelines were followed [30] and the CONSORT diagram was used to describe the flow of participants at each stage of the trial.

### Participants

Sixty-eight individuals with knee OA diagnosed by the Physician as per American College of Rheumatology criteria were recruited for this RCT [31]. The other inclusion criteria were as follows: age 45–66 years and 1–3 radiographic grades on the Kellgren-Lawrence scale [32]. The most symptomatic knee as indicated by the patients was included in the evaluation. The participants were excluded due to a history of knee surgery (*n* = 3), impaired lower limb function due to stiff knee joint (*n* = 1), received an intra-articular injection (*n* = 2) and physical therapy (n = 2) in the last 3 months.

### Interventions

All participants received routine physiotherapy as published previously [33]. The exercise program comprises a combination of closed kinetic chain and open kinetic chain exercises, including straight leg raising, isometric quadriceps, isometric hip adduction, terminal knee extension, semi-squat, and leg press. A previous study has recommended the combination of closed kinetic chain and open kinetic chain exercises for individuals with knee OA [34]. In addition, all participants received ultrasound therapy (1.5 watts/cm2 for 7 min in continuous mode) at the knee joint prior to exercise. In a previous study, this dose of therapeutic ultrasound was found to be safe and effective to reduce pain and disability in individuals with knee OA [35]. Additionally, a recent systematic review suggested that that therapeutic ultrasound is safe and beneficial for reducing pain and improving functions in knee OA [36]. The participants were instructed by a trained Physiotherapist to perform the prescribed exercises 3 days a week for 6 weeks as previously published [Table 1] [33]. All participants were requested to avoid any exercise other than the prescribed program during the trial. Frequent reminders and corrections were given by the therapists who involved in the training to avoid incorrect or any other forms of exercise during the trial.

**Table 1:** Prescribed exercise for all the groups

Exercise	Procedure	Frequency	Intensity	Progression
Isometric quadriceps exercise	Patients lay in a supine position. A rolled up towel was put beneath the knee. They were instructed to maximally activate their thigh muscles in order to straighten their knee and hold the contraction for 5 s.	3 days/week	1 set of 10 repetitions/twice a day	1st week: 1 set 2–3 weeks: 2 sets 3–6 weeks: 3 sets
Straight leg raising (SLR) exercise	Patients lay in a supine position. They were instructed to perform a maximum isometric quadriceps contraction prior to the lifting phase of the exercise. Then they were instructed to lift the leg up to 10 cm above the plinth and hold the contraction during the lifting phase for 10 s.	3 days/week	1 set of 10 repetitions/twice a day	1st week: 1 set 2–3 weeks: 2 sets 3–6 weeks: 3 sets
Isometric hip adduction exercise	Patients lay in a supine position. A small pillow was put between the knees. They were instructed to perform isometric hip adduction exercise while pressing the pillow between the knees and to maintain the adduction with contraction for 5 s.	3 days/week	1 set of 10 repetitions/twice a day	1st week: 1 set 2–3 weeks: 2 sets 3–6 weeks: 3 sets
Terminal knee extension exercise	Patients lay in a supine position. The affected knee is flexed about 30 degrees over a rolled towel. The patients were instructed to extend the knee to zero degree and hold it for 5 s then gradually flex the knee to starting position.	3 days/week	1 set of 10 repetitions/twice a day	1st week: 1 set 2–3 weeks: 2 sets 3–6 weeks: 3 sets
Semi-squat exercise	Patients were asked to stand against the wall and performed semi-squat to 45- degrees flexion at knees and held this position for 30 s.	3 days/week	1 set of 10 repetitions/twice a day	1st week: 1 set 2–3 weeks: 2 sets 3–6 weeks: 3 sets
leg press exercise	Patients were asked to perform leg press exercise on a standard leg press machine. Patients were asked to press the machine to extend the knee to zero degree and hold it for 5 s then gradually flex the knee to starting position.	3 days/week	1 set of 10 repetitions/twice a day	1st week: 1 set 2–3 weeks: 2 sets 3–6 weeks: 3 sets

The participants in the retro walking group completed 10 min of supervised retro walking training with 5-min warm-up and cool-down sessions 3 days a week for 6 weeks at their comfortable walking speed along with a routine physiotherapy as indicated above. The participants were instructed to gradually increase their walking time up to 30 min over the 6-week period, if they consistently obtained a lesser amount of pain e.g. pain scores < 3 on numerical rating scale [37]. In the warm-up and cool-down sessions, the subjects were instructed to perform heel raise exercises, ankle toe movements, and gastrocnemius-soleus and hamstring stretches.

The participants in the forward walking group completed 10 min of supervised forward walking with 5-min warm-up and cool-down sessions 3 days/week for 6 weeks on a flat surface at their comfortable walking speed along with a standard physiotherapy program as mentioned above. The participants were instructed to gradually increase their walking time up to 30 min over the 6-week period. In the warm-up and cool-down sessions, the subjects were instructed to perform ankle toe movements, heel raise exercises, and hamstring and gastrocnemius-soleus stretches.

### Outcomes

All the outcomes were measured by a physical therapist that has been in clinical practice for more than 10 years and has experience of using the outcomes used in this study.

The primary outcome including mean pain and knee function score measured by the numerical rating scale and the Western Ontario and McMaster Universities Osteoarthritis Index (WOMAC), respectively, were analyzed at baseline and week 6. The participants were requested to indicate their level of pain on the numerical rating scale (0–10 scale with 0 indicating no pain and 10 indicating the worst pain). The reliability and validity of the numerical rating scale for measuring musculoskeletal pain has been established [38]. The participants were requested to report their knee function on the WOMAC index, a reliable, valid, and responsive disease-specific instrument [39]. The WOMAC index comprises 24 items including pain (score 0–20), stiffness (scored 0–8), and physical function (scored 0–68), and lower scores indicate better function.

Secondary outcome including mean score of quadriceps muscle strength and timed up and go (TUG) test scores were analyzed at baseline and week 6. The quadriceps muscle strength was measured using the Jamar hydraulic handheld dynamometer (Model, SH5001; SAEHAN, Changwon, South Korea). The participants were instructed to sit at the edge of a treatment table and the knee was maintained at 60 degrees of flexion using a standard goniometer. The dynamometer was placed approximately five centimeters proximal to the distal part of the lateral malleolus. A belt was used around the edge of the treatment table to stabilize the participant’s pelvis [40, 41].

The TUG test was administered as described previously [42]. A firm standard height chair with arms was placed at one end and an object was placed at the other end at a 3-m distance. The test was started with each participant sitting with their back against the chair, arms in their lap, both foot flat on the ground and feet just behind the starting markings on the floor. The participants were instructed as follows: “On the word ‘go,’ stand up, walk comfortably and safely to the object at the end on the floor, walk around the object, come back, and sit all the way back in your chair.” Timing was started on the word “go” and ended when the participant was seated with their back resting against the chair [43]. A practice trial was given first, followed by two recorded trials. The mean of the two recorded trials was used in the analysis.

Adverse effects were defined as perception of increased knee pain due to trial protocol lasting ≥2 days or the participant had consulted Physician or took medications to relieve symptoms. Falls or injuries to other body parts during trial were considered adverse effects.

All measurements were performed at baseline (week 0) and the end of the intervention (week 6). A senior physical therapist blinded to the group assignments was responsible for recording the measurements.

### Sample size

The statistical software Statmate version 2 (GraphPad Software, Inc., CA, USA) was used to determine the required sample size using the primary outcome variable NRS scores for pain, with a power of 80% and a significance level of 0.05 (two-tailed). The standard deviation (SD) was calculated using the data of previous published study on the effects of retro-walking [44]. A clinically important difference between groups of 1.08 in NRS score (SD = 1.19). The sample size calculation yields 20 participants in each group (a total of 60 participants). To allow for a potential drop-out of 15%, we recruited a minimum of 68 participants.

### Data analysis

SPSS software version 22 (SPSS, Chicago, IL, USA) was used for the statistical analysis. Mean and standard deviation are reported for all results. Baseline scores of all outcome measures and the demographic data were presented to assess baseline comparability of the treatment groups. Descriptive data were reported for each group as the mean change in the outcome measures at baseline and at the end of the trial. Data normality was tested using the Shapiro-Wilk test. A 3 × 2 two-way analysis of variance was used to compare the effects of group (retro walking, forward walking, and control) and timing (pre- and post-test). If interactions were detected, a post hoc analysis with Bonferroni adjustment was employed. The level of significance was set at *p* < 0.05.

## Results

### Enrollment and follow-up

**Fig. 1:**
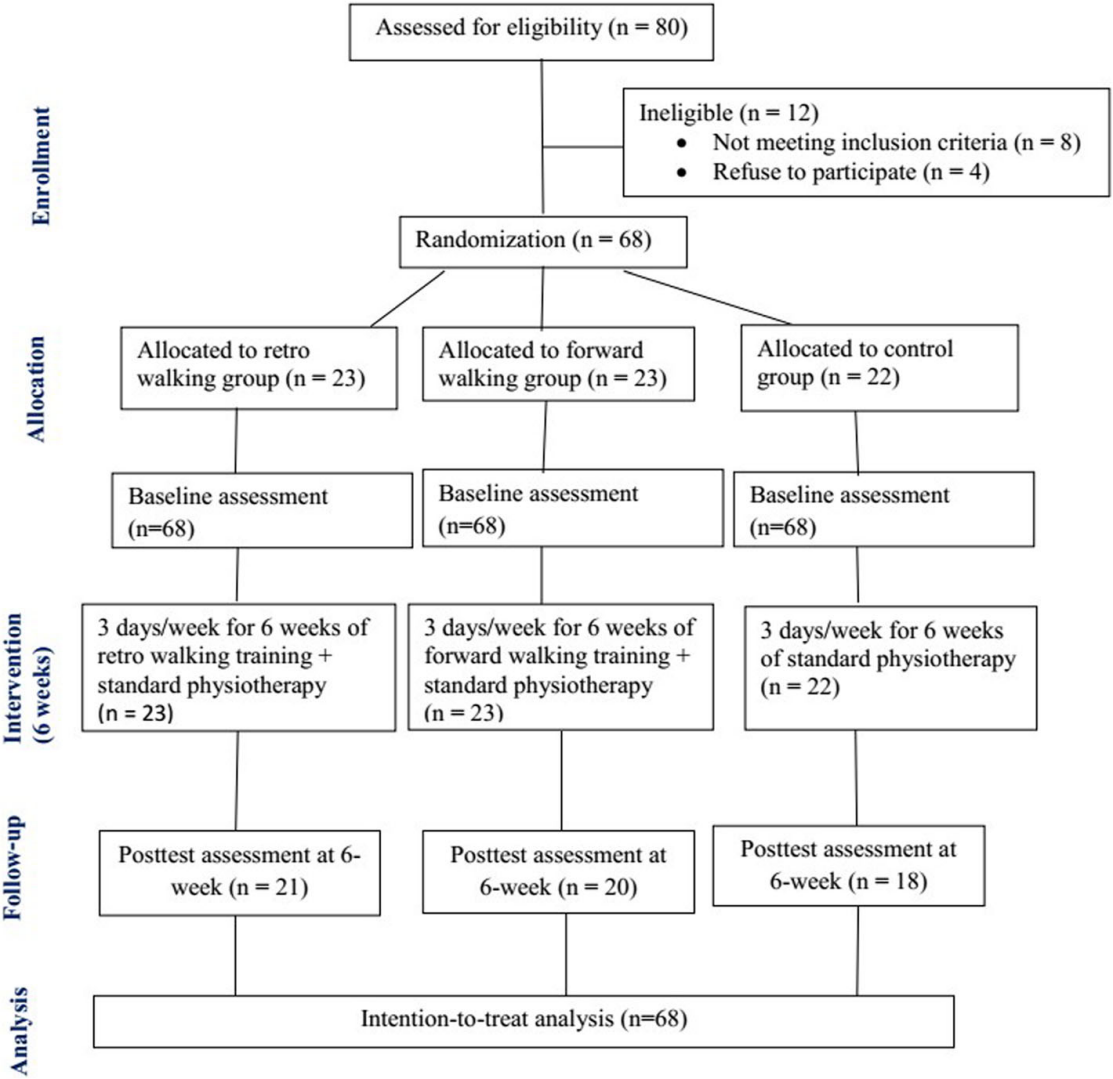
Flow of participants through each stage of the randomized trial

In total, 68 subjects participated in this 6-week randomized controlled trial (Fig. 1). The completion rates of the primary and secondary outcome measures at week 6 were 91, 87, and 82% in the retro walking, forward walking, and control groups, respectively. All 68 subjects were included in the intention-to-treat analysis to control drop out data. For missing data, the last observation carried forward method, in which the last available data for each participant at the time point prior to withdrawal from the study was retained in the analysis [45, 46].

### Patient characteristics

Baseline scores of all outcome measures and the demographic data among the three groups showed non-significant differences (Table 2). Fifty-six percent of the participants were women. Baseline scores for all the participants were 5.9 (numerical rating scale), 53.3 (WOMAC), 10.8 kg (quadriceps muscle strength) and 9.4 s (TUG).

**Table 2:** Participant characteristics at baseline

	All	Retro walking group	Forward walking group	Control group	*p* value
Mean age (min-max), years	55.6 (45–66)	54.6 (45–66)	55.3 (47–65)	56. 8 (46–66)	.471
Gender (male/female), n	30/38	12/11	9/14	9/13	.639
Mean weight (min-max), kg	62.8 (40–100)	63.4 (40–100)	62.6 (49–78)	62.3 (46–78)	.921
Mean height (min-max), m	1.5 (1.4–1.7)	1.5 (1.4–1.7)	1.5 (1.4–1.7)	1.5 (1.4–1.7)	.788
Mean BMI (min-max), kg/m^2^	26.1 (14.7–33.3)	26.2 (14.7–33.3)	26.1 (18.1–30.5)	26.1 (20.4–30.1)	.952
Mean (SD) NRS	5.9 (0.8)	5.7 (0.8)	6.1 (0.7)	5.8 (0.8)	.197
Mean (SD) WOMAC, %	53.3 (3.2)	52.9 (3.3)	53.3 (3.1)	53.8 (3.3)	.639
Mean (SD) Quadriceps muscle strength, kg	10.8 (1.9)	11.1 (2.2)	11.04 (1.6)	10.2 (1.9)	.235
Mean (SD) TUG, sec	9.4 (0.3)	9.4 (0.4)	9.3 (0.3)	9.3 (0.3)	.599

*NRS* numerical rating scale, *WOMAC* Western Ontario and McMaster Universities Osteoarthritis Index, *n* number of participants, *TUG* timed up and go test

### Outcomes

Tables 3 detail the outcome assessment at 6 weeks at the end of trial completion. The intention-to-treat analysis showed that the retro walking group had a significantly greater reduction of pain intensity (mean changes, 1.8 versus 1; *p* = 0.01) and functional disability (mean changes, 4.8 versus 2.2; *p* = 0.008) than the control group. Similarly, the retro walking group had a greater improvement in the quadriceps muscle strength (mean changes, 1.7 kg versus 0.7 kg; p = 0.008) and the timed up and go test (mean changes, 0.6 s versus 0.1 s; *p* = 0.003) than the control group. In addition, the retro- versus forward-walking groups or the forward walking versus control groups showed non-significant differences in all the outcomes (*p* > 0.05).

**Table 3:** Outcome measures at six weeks after completion of trial in retro walking, forward walking, and control groups (Intention-to-treat analysis, *n* = 68)

Variables	Retro walking (RW) group			Forward walking (FW) group			Control group (CG)			ANOVA		Post hoc analysis (Bonferroni)		
	Mean (SD)	95% CI	Mean changes	Mean (SD)	95% CI	Mean changes	Mean (SD)	95% CI	Mean changes	F	p	RW vs. FW	RW vs. CG	FW vs. CG
NRS	3.9 (1.1)	3.4, 4.3	1.8	4.6 (1.3)	3.9, 5.2	1.5	4.8 (0.8)	4.5, 5.2	1	4.584	0.014*	0.10	0.01†	0.94
WOMAC (%)	48.1 (3.4)	46.6, 49.6	4.8	50.2 (4.2)	48.4, 52.1	3.1	51.6 (3.6)	49.9, 53.2	2.2	4.941	0.010*	0.16	0.008†	0.74
Quadriceps muscle strength (kg)	12.8 (1.9)	11.8, 13.6	1.7	12 (1.7)	11.3, 12.7	0.96	10.9 (2.3)	9.9, 11.9	0.7	4.854	0.011*	0.549	0.008†	0.241
TUG (sec)	8.8 (0.5)	8.6, 9.1	0.6	9.1 (0.3)	8.9, 9.3	0.2	9.2 (0.3)	9.1, 9.4	0.1	6.171	0.004*	0.06	0.003†	0.89

*ANOVA* analysis of variance, *NRS* numerical rating scale, *WOMAC* Western Ontario and McMaster Universities Osteoarthritis Index, *TUG* timed up and go test, *CI* Confidence interval, *SD* Standard deviation

*p < 0.05. †*p* (adjusted) < 0.017

### Serious adverse events

No serious adverse events were reported for this trial. In few patients (*n* = 3), mild increase of pain was reported in the forward walking group. Walking time and exercise load was adjusted to reduce pain accordingly.

## Discussion

The present randomized, controlled trial aimed to compare the effects of retro or forward walking programs on pain, function, quadriceps muscle strength, and performance in people with knee OA. The results of the present study indicated that the retro walking program is effective in reducing pain and improving function, quadriceps muscle strength, and performance after 6 weeks in people with knee OA. However, there were no significant differences in any outcomes between retro and forward walking or forward walking and the traditional physiotherapy program. Biomechanically, muscles around ankle and knee reversed their action during retro-walking. In retro-walking, knee gives the primary power producer with co-contraction of quadriceps and hamstring and ankle plantar flexors works as shock absorber [11]. In retro-walking, shear force at knee joint directed anteriorly whereas it moves posteriorly in forward walking [47]. Additionally, retro-walking causes significantly reduced patellar compressive force than forward walking [48]. It is well known that physical exercise in the form of walking is cost-effective, accessible, and effective in reducing cardio-vascular disease [49], obesity [50, 51], and symptoms of depression [52, 53]. Additionally, walking is the most common form of physical activity in the United Kingdom and the United States [54, 55].

Similarly, a recent report suggested significantly improved function after 3 weeks of retro walking in addition to the routine physiotherapy in person with knee OA [26]. Other studies reported reduced pain and disability after a combined walking program and weight training and improved postural stability following an aerobic walking and long-term weight training programs in person with knee OA [56, 57]. In contrast to current study, a 3-month RCT investigating the effectiveness of a walking program (forward walking) and home exercise in person with knee OA indicated significant improvements of 51–55% on the WOMAC pain subscale and of 57% on the WOMAC physical function subscale compared with controls [58]. However, in the current study, the control group received traditional physiotherapy program. This could be the reason that present study did not find statistically significant difference in any outcomes between forward walking versus control groups. Previous study indicated that the retro-walking reduces eccentric activity of the quadriceps, while isometric and concentric quadriceps activity was maintained [24]. This is one of the advantages of retro walking over forward walking. Reduced eccentric activity of quadriceps will results decrease compressive force at knee joint, therefore, pain intensity at the knee will be reduced. In contrast to present study, previous study indicated increased quadriceps strength after a backward running compared with forward running program [22]. However, later study included only healthy participants. Another study reported a negative effect of a walking program in person with knee OA [29]. However, only obese women were participated in this study. Therefore, the weight-bearing nature of walking might cause increased pain intensity in these subjects. In contrast to current study, a phase II RCT reported no changes in knee pain following a 12-week walking program in person with severe knee OA [28]. However, there was a methodological difference between former and current study. The current study excluded subjects with severe knee OA. Inclusion of patients with severe knee OA in the previous study could be the reason that the previous study did not find any substantial changes in knee pain following a 12-week walking program.

The present study demonstrated that the retro walking program more effectively improved performance as measured by TUG test than the forward walking or traditional physiotherapy program. A previous study reported that a home-based pedometer-driven walking program improved walking performance in individuals with knee OA [39]. More recently, immediate improvements in knee symptoms and mobility-related restrictions following personalized gait training was reported in people with symptomatic knee OA; however, these improvements were not maintained at 6 or 12 months of follow-up [59]. In addition, a positive effect of walking program and exercise on health-related quality of life in person with knee OA was noted [60].

A recent phase I trial indicated that 70 min of supervised moderate-intensity walking per week is safe and feasible for individuals with severe knee OA [27]. In the present study, the walking program length was gradually increased from 30 min per week to 90 min per week over the 6-week period to prevent any symptom worsening. Another study reported that walking in a pre-intervention program is feasible, safe, and more effective than a mixed pre-operative program in people with knee OA [61]. Similarly, the present study reported no adverse events. In addition, a recent systematic review indicated no significant difference in exercise intensity, e.g., high- versus low-intensity aerobic or resistance exercises in people with knee OA [62].

The results of current study indicate a large significant effect of retro walking compared to control group. A previous systematic review demonstrated a larger effect size for exercise than no exercise for reducing pain in patients with knee OA [63]. Further sub group analysis indicated a larger effect size for open kinetic chain exercise than closed kinetic chain exercise and aerobic exercise [63]. Another systematic review reported moderated effect size for aerobic, resistance, and performance exercise in reducing pain in patients with knee OA [64].

The present study had some potential limitations. First, due to lack of power with three small groups, this study might have been underpowered to detect a significant effect of each treatment group. A long-term follow-up was not included in the present study due to a poor history of patient follow-up in the current hospital setting. Long term trial might bring greater changes in the outcomes and therefore, we could see significant difference between retro- and forward-walking programs. In addition, only subjects aged 45–66 years participated. Impaired cognitive function and severe pain were reported in patients ≥70 years with knee OA [65]. Furthermore, another study reported a relationship between poor physical function and worse cognitive function in elderly individuals with knee OA [33]. Moreover, a risk of falls was increased in elderly individuals with knee OA as reported previously [66]. In the future, we should include both younger and older patients to see a comprehensive clinical picture of these exercise program.

## Conclusions

In conclusion, the present study indicated that a 6-week retro walking program compared with forward walking or control groups resulted in greater reduction in pain and functional disability and improved quadriceps muscle strength and performance in individuals with knee OA. Since, retro-walking has many advantages over forward walking, we believe that society will utilize these forms of exercise in their daily life to improve their quality of life. After some training, people can easily be able to do the retro-walking in the public parks.

## Abbreviations

OA: Osteoarthritis
TUG: timed up and go test
WOMAC: Western Ontario and McMaster Universities Osteoarthritis Index

## References

[1] Cross M, Smith E, Hoy D, Nolte S, Ackerman I, Fransen M (2014). The global burden of hip and knee osteoarthritis: estimates from the global burden of disease 2010 study. Ann Rheum Dis.

[2] GBD 2016 Disease and Injury Incidence and Prevalence Collaborators (2017). Global, regional, and national incidence, prevalence, and years lived with disability for 328 diseases and injuries for 195 countries, 1990–2016: a systematic analysis for the Global Burden of Disease Study 2016. Lancet.

[3] Neogi T (2013). The epidemiology and impact of pain in osteoarthritis. Osteoarthr Cartil.

[4] Yázigi F, Espanha M, Marques A, Teles J, Teixeira P (2018). Predictors of walking capacity in obese adults with knee osteoarthritis. Acta Reumatol Port.

[5] Hunter DJ, Schofield D, Callander E (2014). The individual and socioeconomic impact of osteoarthritis. Nat Rev Rheumatol.

[6] Wang H, Bai J, He B, Hu X, Liu D (2016). Osteoarthritis and the risk of cardiovascular disease: a meta-analysis of observational studies. Sci Rep.

[7] Yokota RT, Van der Heyden J, Demarest S, Tafforeau J, Nusselder WJ, Deboosere P (2015). Contribution of chronic diseases to the mild and severe disability burden in Belgium. Arch Public Health.

[8] van der Esch M, Knoop J, van der Leeden M, Roorda LD, Lems WF, Knol DL (2015). Clinical phenotypes in patients with knee osteoarthritis: a study in the Amsterdam osteoarthritis cohort. Osteoarthr Cartil.

[9] Jönsson T, Ekvall Hansson E, Thorstensson CA, Eek F, Bergman P, Dahlberg LE (2018). The effect of education and supervised exercise on physical activity, pain, quality of life and self-efficacy - an intervention study with a reference group. BMC Musculoskelet Disord.

[10] Hurley M, Dickson K, Hallett R, Grant R, Hauari H, Walsh N (2018). Exercise interventions and patient beliefs for people with hip, knee or hip and knee osteoarthritis: a mixed method review. Cochrane Database Syst Rev.

[11] Chen LY, Su FC, Chiang PY (2000). Kinematic and EMG analysis of backward walking on treadmill. Conf Proc IEEE Eng Med Biol Soc.

[12] Cipriani DJ, Armstrong CW, Gaul S (1995). Backward walking at three levels of treadmill inclination: an electromyographic and kinematic analysis. J Orthop Sports Phys Ther.

[13] Jan MH, Tang PF, Lin JJ, Tseng SC, Lin YF, Lin DH (2008). Efficacy of a target-matching foot-stepping exercise on proprioception and function in patients with knee osteoarthritis. J Orthop Sports Phys Ther.

[14] Ojoawo AO, Olaogun MO, Hassan MA (2016). Comparative effects of proprioceptive and isometric exercises on pain intensity and difficulty in patients with knee osteoarthritis: a randomized control study. Technol Health Care.

[15] Takacs J, Krowchuk NM, Garland SJ, Carpenter MG, Hunt MA (2017). Dynamic balance training improves physical function in individuals with knee osteoarthritis: a pilot randomized controlled trial. Arch Phys Med Rehabil.

[16] McAlindon TE, Bannuru RR, Sullivan MC, Arden NK, Berenbaum F, Bierma-Zeinstra SM (2014). OARSI guidelines for the non-surgical management of knee osteoarthritis. Osteoarthr Cartil.

[17] Egan BA, Mentes JC (2010). Benefits of physical activity for knee osteoarthritis: a brief review. J Gerontol Nurs.

[18] Hochberg MC, Altman RD, April KT, Benkhalti M, Guyatt G, McGowan J (2012). American College of Rheumatology 2012 recommendations for the use of non-pharmacologic and pharmacologic therapies in osteoarthritis of the hand, hip, and knee. Arthritis Care Res.

[19] Roddy E, Zhang W, Doherty M (2005). Aerobic walking or strengthening exercise for osteoarthritis of the knee? A systematic review. Ann Rheum Dis.

[20] Kovar PA, Allegrante JP, MacKenzie CR, Peterson MG, Gutin B, Charlson ME (1992). Supervised fitness walking in patients with osteoarthritis of the knee. A randomized, controlled trial. Ann Intern Med.

[21] Uthoff A, Oliver J, Cronin J, Harrison C, Winwood P (2018). A new direction to athletic performance: understanding the acute and longitudinal responses to backward running. Sports Med.

[22] Threkeld AJ, Horn TS, Wojtowicz GM, Rooney JG, Shapiro R (1989). Kinematics, ground reaction force, and muscle balance produced by backward running. J Orthop Sports Phys Ther.

[23] Zhang M, Liu A, Jiang L, Pang J, Guo H, Chen B, Zhao Y, Chen D, Zhan H (2015). The biomechanical effects of backward walking on the knee: a new method for releasing the joint loading. Osteoarthr Cartil.

[24] Masumoto K, Soucy MT, Bailey JP, Mercer JA (2017). Muscle activity during backward and forward running with body weight support. Hum Mov Sci.

[25] Loew L, Brosseau L, Kenny GP, Durand-Bush N, Poitras S, De Angelis G (2017). An evidence-based walking program among older people with knee osteoarthritis: the PEP (participant exercise preference) pilot randomized controlled trial. Clin Rheumatol.

[26] Gondhalekar GA, Deo MV (2013). Retro walking as an adjunct to conventional treatment versus conventional treatment alone on pain and disability in patients with acute exacerbation of chronic knee osteoarthritis: a randomized clinical trial. N Am J Med Sci.

[27] Wallis JA, Webster KE, Levinger P, Singh PJ, Fong C, Taylor NF (2015). The maximum tolerated dose of walking for people with severe osteoarthritis of the knee: a phase I trial. Osteoarthr Cartil.

[28] Wallis JA, Webster KE, Levinger P, Singh PJ, Fong C, Taylor NF (2017). A walking program for people with severe knee osteoarthritis did not reduce pain but may have benefits for cardiovascular health: a phase II randomised controlled trial. Osteoarthr Cartil.

[29] Toda Y (2001). The effect of energy restriction, walking and exercise on lower extremity lean body mass in obese women with osteoarthritis of the knee. J Orthop Sci.

[30] Boutron I, Altman DG, Moher D, Schulz KF (2017). Ravaud P; CONSORT NPT Group. CONSORT statement for randomized trials of nonpharmacologic treatments: a 2017 update and a CONSORT extension for nonpharmacologic trial abstracts. Ann Intern Med.

[31] Damen J, van Rijn RM, Emans PJ, Hilberdink WKHA, Wesseling J, Oei EHG (2019). Prevalence and development of hip and knee osteoarthritis according to American College of Rheumatology criteria in the CHECK cohort. Arthritis Res Ther.

[32] Kellgren JH, Lawrence JS (1987). Radiologic assessment of osteoarthritis. Ann Rheum Dis.

[33] Anwer S, Alghadir A (2014). Effect of isometric quadriceps exercise on muscle strength, pain, and function in patients with knee osteoarthritis: a randomized controlled study. J Phys Ther Sci.

[34] Anwer S, Alghadir A, Brismée JM (2016). Effect of home exercise program in patients with knee osteoarthritis: a systematic review and meta-analysis. J Geriatr Phys Ther.

[35] Yeğin T, Altan L, Kasapoğlu AM (2017). The effect of therapeutic ultrasound on pain and physical function in patients with knee osteoarthritis. Ultrasound Med Biol.

[36] Zhang C, Xie Y, Luo X, Ji Q, Lu C, He C (2016). Effects of therapeutic ultrasound on pain, physical functions and safety outcomes in patients with knee osteoarthritis: a systematic review and meta-analysis. Clin Rehabil.

[37] Bruce-Brand RA, Walls RJ, Ong JC, Emerson BS, O'Byrne JM, Moyna NM (2012). Effects of home-based resistance training and neuromuscular electrical stimulation in knee osteoarthritis: a randomized controlled trial. BMC Musculoskelet Disord.

[38] Alghadir AH, Anwer S, Iqbal A, Iqbal ZA (2018). Test-retest reliability, validity, and minimum detectable change of visual analog, numerical rating, and verbal rating scales for measurement of osteoarthritic knee pain. J Pain Res.

[39] Ayala A, Bilbao A, Garcia-Perez S, Escobar A, Forjaz MJ (2018). Scale invariance and longitudinal stability of the physical functioning Western Ontario and MacMaster universities osteoarthritis index using the Rasch model. Rheumatol Int.

[40] Matsuse H, Hashida R, Takano Y, Omoto M, Nago T, Bekki M (2017). Walking exercise simultaneously combined with neuromuscular electrical stimulation of antagonist’s resistance improved muscle strength, physical function, and knee pain in symptomatic knee osteoarthritis: a single-arm study. J Strength Cond Res.

[41] Talbot LA, Gaines JM, Huynh TN, Metter EJ (2003). A home-based pedometer-driven walking program to increase physical activity in older adults with osteoarthritis of the knee: a preliminary study. J Am Geriatr Soc.

[42] Podsiadlo D, Richardson S (1991). The timed “up & go”: a test of basic functional mobility for frail elderly persons. J Am Geriatr Soc.

[43] Alghadir A, Anwer S (2016). Effect of retro and forward walking on quadriceps muscle strength, pain, function, and mobility in patients with knee osteoarthritis: a protocol for a randomized controlled trial. BMC Musculoskelet Disord.

[44] Rathi M, Palekar T, Varghese A (2014). Efficacy of backward walking on patients with osteoarthritis of knee on quadriceps strength, pain, and physical functions. Indian J Physiother Occup Ther.

[45] Dossing A, Tarp S, Furst DE, Gluud C, Wells GA, Beyene J (2016). Modified intention-to-treat analysis did not bias trial results. J Clin Epidemiol.

[46] Joseph R, Sim J, Ogollah R, Lewis M (2015). A systematic review finds variable use of the intention-to-treat principle in musculoskeletal randomized controlled trials with missing data. J Clin Epidemiol.

[47] Kumar TR, Ashraf M (2009). The effect of backward walking treadmill training on kinematics of the trunk and lower limbs. Serb J Sports Sci.

[48] Van der Esch M, Holla JF, van der Leeden M, Knol DL, Lems WF, Roorda LD (2014). Decrease of muscle strength is associated with increase of activity limitations in early knee osteoarthritis: 3-year results from the cohort hip and cohort knee study. Arch Phys Med Rehabil.

[49] Boone-Heinonen J, Evenson KR, Taber DR, Gordon-Larsen P (2009). Walking for prevention of cardiovascular disease in men and women: a systematic review of observational studies. Obes Rev.

[50] Morabia A, Costanza MC (2004). Does walking 15 minutes a day keep the obesity epidemic away? Simulation of the efficacy of a population wide campaign. Am J Public Health.

[51] Pucher J, Buehler R, Bassett D, Dannenberg A (2010). Walking and cycling to health: a comparative analysis of city, state, and international data. Am J Public Health.

[52] Robertson R, Robertson A, Jepson R, Maxwell M (2012). Walking for depression or depressive symptoms: a systematic review and meta-analysis. Ment Health Phys Act.

[53] Armstrong K, Edwards H (2004). The effectiveness of a pram-walking exercise programme in reducing depressive symptomatology for postnatal women. Int J Nurs Pract.

[54] Craig R, Mindell J, Hirani V (2009). Health Survey for England 2008: Volume 1 Physical Activity and Fitness.

[55] Centers for Disease Control and Prevention. More People Walk to Better Health. [(accessed on 22 January 2019)]; Available online: https://www.cdc.gov/vitalsigns/walking/index.html

[56] Messier SP, Royer TD, Craven TE, O’Toole ML, Burns R, Ettinger WH (2000). Long-term exercise and its effect on balance in older, osteoarthritic adults: results from the fitness, arthritis, and seniors trial (FAST). J Am Geriatr Soc.

[57] Messier SP, Loeser RF, Mitchell MN, Valle G, Morgan TP, Rejeski WJ (2000). Exercise and weight loss in obese older adults with knee osteoarthritis: a preliminary study. J Am Geriatr Soc.

[58] Evcik D, Sonel B (2002). Effectiveness of a home-based exercise therapy and walking program on osteoarthritis of the knee. Rheumatol Int.

[59] Segal NA, Glass NA, Teran-Yengle P, Singh B, Wallace RB, Yack HJ (2015). Intensive gait training for older adults with symptomatic knee osteoarthritis. Am J Phys Med Rehabil.

[60] Dias RC, Dias JM, Ramos LR (2003). Impact of an exercise and walking protocol on quality of life for elderly people with OA of the knee. Physiother Res Int.

[61] Wallis JA, Webster KE, Levinger P, Fong C, Taylor NF (2014). A preoperative group rehabilitation programme provided limited benefit for people with severe hip and knee osteoarthritis. Disabil Rehabil.

[62] Smith T, Kirby E, Davies L (2014). A systematic review to determine the optimal type and dosage of land-based exercises for treating knee osteoarthritis. Phys Ther Rev.

[63] Tanaka R, Ozawa J, Kito N, Moriyama H (2013). Efficacy of strengthening or aerobic exercise on pain relief in people with knee osteoarthritis: a systematic review and meta-analysis of randomized controlled trials. Clin Rehabil.

[64] Juhl C, Christensen R, Roos EM, Zhang W, Lund H (2014). Impact of exercise type and dose on pain and disability in knee osteoarthritis: a systematic review and meta-regression analysis of randomized controlled trials. Arthritis Rheumatol.

[65] Morone NE, Abebe KZ, Morrow LA, Weiner DK (2014). Pain and decreased cognitive function negatively impact physical functioning in older adults with knee osteoarthritis. Pain Med.

[66] Muraki S, Akune T, Oka H, En-Yo Y, Yoshida M, Nakamura K (2011). Prevalence of falls and the association with knee osteoarthritis and lumbar spondylosis as well as knee and lower back pain in Japanese men and women. Arthritis Care Res (Hoboken).

